# Age–Period–Cohort Analysis of Long Trend of Mortality for Stroke and Subtypes Attributed to High SBP in Chinese Adults

**DOI:** 10.3389/fneur.2022.710744

**Published:** 2022-03-09

**Authors:** Yudiyang Ma, Jinhong Cao, Sumaira Mubarik, Jianjun Bai, Donghui Yang, Yudi Zhao, Qian Hu, Chuanhua Yu

**Affiliations:** ^1^Department of Epidemiology and Biostatistics, School of Public Health, Wuhan University, Wuhan, China; ^2^Department of Public Health, Tongji Medical College, Huazhong University of Science and Technology, Wuhan, China

**Keywords:** stroke, subtypes, mortality, Chinese adults, age–period–cohort model

## Abstract

Stroke has been found as the leading cause of death in China, and high systolic blood pressure (SBP) has been indicated as a critical risk factor of stroke mortality. Accordingly, in this article, the aims were to investigate the long-term trends of mortality in terms of stroke and subtypes arising from high SBP stratified by age and gender among Chinese adults. The data of this article originated from the global burden of disease (GBD) study database. The age, period, and cohort effects were examined with the age–period–cohort model (APC). The age-standardized mortality of stroke attributed to high SBP in China has been significantly declining from 1990 to 2019. As indicated by the result of APC analysis, substantially rising age effects have been exerted on stroke and subtypes from 25 to 84 years of both genders, whereas the age effect on women increased less rapidly than that on men. As opposed to the above result, the period and cohort effects were reported to show similar monotonous decrease trends, and stroke of women more significantly declined than that of men (significantly with *p* < 0.05 for all). All types of stroke mortality arising from high SBP were indicated to change. The risk of death was identified to be most remarkably reduced in high SBP-attributable subarachnoid hemorrhage (SAH), whereas intracerebral hemorrhage (ICH) and ischemic stroke (IS) decreased at approximately the same rate. People born in the later birth cohorts or living in recent periods had a lower risk of stroke death, whereas men and elder groups were considered as the high-risk populations for stroke mortality due to high SBP. Although the stroke mortality relating to high SBP in China was declining, ICH and IS would continue to be the first and second lethal subtypes of stroke. In contrast to the above finding, SAH accounted for the minimum proportion of deaths and the maximum reduction in period and cohort effects. Thus, it is of high significance to introduce advanced hypertension control technology and knowledge regarding healthy lifestyles.

## Introduction

Stroke, a noncommunicable disease, was reported as the second leading cause of death in 2019, with about 101 million cases and 6.55 million deaths worldwide (1). Pathologically, stroke can generally fall into three types, which include ischemic stroke (IS), intracerebral hemorrhage (ICH), and subarachnoid hemorrhage (SAH) (2). Chinese populations have been reported with a higher proportion of ICH and also a different distribution of IS and SAH, as compared to western populations (3, 4). As indicated by the latest study related to burden of disease (5), high systolic blood pressure (SBP) has always been the leading risk factor for the burden of stroke since 2010. In contrast to diastolic blood pressure or pulse pressure, SBP of over 110 mm Hg acts as a leading and suggestive risk factor for global disease burden (6). Moreover, it is a vital predictor and a modifiable factor for most types of cardiovascular diseases or diabetes (7, 8). As highlighted by numerous valuable studies, blood pressure control has been the most effective intervention for stroke prevention (9, 10). However, in China, high SBP is characterized by a spatiotemporal pattern, which implies that it is difficult to control completely since the process from diagnosis to treatment is relatively long; not all patients suffering from abnormal SBP can be detected (11). Among Chinese adults aged 35–75 years, 30% were undiagnosed with high SBP, and the cure or control rate has been <50% (12). More seriously, the lack of appropriate therapies for stroke patients with high SBP increased the risk of death by nearly 3.1 times for men and 2.9 times for women (13).

By investigating the trend of stroke mortality attributed to high SBP by the age–period–cohort model (APC) in China, a novel insight can be gained into the stroke burden at nation level. Furthermore, rare studies have explored the mortality of high SBP-attributable stroke between time scale and age or cohort groups and separately assessed the temporal trend and disparity of IS, ICH, and SAH. Previous studies have only focused on one type of stroke solely and reported changes in age-specific mortality, whereas the above studies failed to decompose cohort effects covered by long-term trends (14). In the above circumstance, this article primarily estimated stroke and its subtype mortality arising from high SBP over the past three decades in China. Independent effects arising from age, period, birth cohort, and temporal trends were analyzed using the APC with an intrinsic estimator (IE) algorithm. The above analysis is of high significance to identify gaps of all types of stroke burden between different age and cohort groups during the nation's development, which can help policymakers ensure targeted medical schemes meet the needs of the correspondent groups.

## Materials and Methods

The global burden of disease (GBD) 2019, contributed by the Institute for Health Metrics and Evaluation (IHME), aims to quantify the comparative magnitude of health loss arising from diseases, injuries, and risk factors by age, sex, and geographies for specific points in a series of periods (15). All anonymized data are publicly available at the IHME website and accessible online (http://ghdx.healthdata.org/gbd-results-tool). The informed consent was reviewed, and this study gained the approval from the University of Washington Institutional Review Board. DisMod-MR 2.1, a Bayesian metaregression tool, was used as the primary method of estimation to ensure consistency between rates of incidence, prevalence, remission, and cause of death for the respective condition, and a comprehensive annual estimation was conducted for global, regional, and national incidence, prevalence, mortality for causes of death and risk factors in 204 nations and territories from 1990 to 2019 (5). The GBD approach has an advantage that consistent methods are adopted to critically appraise available information on the respective condition, which promotes the information to be comparable and systematic, adopted to estimate results from nations with incomplete data, and also adopted to report the burden of disease with standardized metrics.

### Data Sources

The attributable burden of stroke data in China and India originated from the GBD 2019. Stroke was diagnosed and defined in accordance with the WHO clinical criteria and the International Statistical Classification of Diseases. Stroke subtypes (e.g., IS, ICH, and SAH events) were classified based on the tenth revision of the International Classification of Diseases and Injuries (ICD-10). In general, the original data of stroke mortality in China population originated from the Cause of Death Reporting System of the Chinese Center for Disease Control and Prevention (CDC), and Disease Surveillance Points (DSPs), which could be nationally representative (16). Indian stroke mortality database comprised vital registration (VR), verbal autopsy (VA), registry, survey, police, and also surveillance data (17).

In the GBD study, high SBP was defined as individual with SBP of over 115 mmHg (5). The data originated from the standard multination survey series [e.g., Demographic and Health Surveys (DHS), Living Standards Measurement Surveys (LSMS), Multiple Indicator Cluster Surveys (MICS), and World Health Surveys (WHS)] and also country-specific survey series [e.g., China Monitoring Survey (5)]. Population attributable fraction (PAF) was defined as if the exposure of a certain risk factor declined to the theoretical minimum exposure level in a certain population, the proportion of related diseases or deaths in the population would reduce (18). Estimates of stroke mortality attributed to high SBP were equated with the total number of stroke deaths multiplied by the PAF for the risk-outcome pair for a given age, gender, location, and also year.


$$\begin{array}{l} PAF=\;\frac{\sum_{i}^{n}P_{i}\left(RR_{i}-1\right)}{\sum_{i}^{n}P_{i}\left(RR_{i}-1\right)+1}\; \end{array}$$

where *P*_*i*_ denotes the percentage of the population exposed to level *i* of high SBP; *n* represents the total number of exposure level. *RR*_*i*_ expresses the relative risk at exposure level *i*, estimated as the integrated exposure response function of exposure based on 81 published systematic reviews. The specific methods were outlined in a previous study (5).

Attributable deaths (ADs) were determined by multiplying the PAF and the number of the deaths for stroke (N) (19):


$$\begin{array}{l} AD=PAF^{*}N\; \end{array}$$

The age-standardized rate of attributable deaths caused by high SBP was determined by the world standard population (20). The person-years were the product of the number of years times the number of members of standard population from Chinese CDC who have been affected by stroke and its subtypes (16). For the specific calculation method, please refer to see http://ghdx.healthdata.org/help/ghdx-help. The population, mortality number, ASMRs data of high SBP-attributable stroke, and the subtypes of diseases in China originated from GBD 2019. Ethical approval was not required by this article since human subjects were not directly involved.

### Statistical Analyses

According to the assumption of the APC model, the observed number of diseases had a Poisson distribution, and the mortality could be a multiplicative function of age, cohort, and period. Accordingly, the logarithm of the rates is an additive function of the parameters. Age effects revealed the different risks of a variety of outcomes in different periods of life; period effects indicate population-wide exposure at a circumscribed point in time, and cohort effects basically represent the disparities in risk across birth cohorts.

Since an individual's birth cohort is determined using the time period of death and the individual's death age (i.e., birth cohort = period – age), the exact linear dependence of the regression variables leads to the identifiable problem. An APC analysis was conducted using the R-based web tool from the US National Cancer Institute to address the identifiable problem (21). The website (http://analysistools.nci.nih.gov/apc/help.html#example) elucidates the arrangement of age–period data arrays and the method of the R-based web tool application, and GitHub (https://github.com/CBIIT/nci-webtools-dceg-age-period-cohort) details the method's core operation code. Numerous useful functions can be estimated if age, period, and cohort trends are orthogonally decomposed into their linear and nonlinear parts (22). Four parameters adopted in the process of analysis in this article are presented below: (1) net drift, the log-linear trend of the annual percent change in the mortality over the period, estimates the average annual percentage change in the mortality logarithm with the period and cohort effect adjustments; (2) local drift represents the average annual percentage changes in mortality over time across a range of age groups; (3) longitudinal age curve reveals the fitted longitudinal age-specific rates in the reference cohort adjusted for period deviations; (4) the period (or cohort) rate ratios (RRs) would be the relative risk adjusted for age and nonlinear effects in a period (or cohort) vs. the reference. For APC analysis, the mortality and population data were arranged into consecutive 5-year periods (1990–2019) and successive 5-year age intervals from 25–29 to 80–84 years. Since the different choice of reference groups in APC model would lead to different results, the reference groups were set as indicated by Rosenberg et al. (21). The central age group, period, or birth cohort in the respective interval were defined as the reference in all APC analyses. In case of an even number of categories, the reference value was set to the lower of the two central values (23, 24). Wald chi-square tests were performed for the significance of the above functions. A general linear model was employed to examine the interaction effect between gender and birth cohorts or the significance of the slope of relative risks for period and cohort effects. All statistical tests are two-sided.

## Results

Stroke mortality attributable to metabolic risks is correlated with six metabolic risk factors (high body mass index [BMI], high fasting plasma glucose [FPG], high SBP, high low-density lipoprotein [LDL] cholesterol, kidney dysfunctions, and other metabolic risks) in the GBD database. High SBP-attributable deaths account for dominance among all stroke deaths caused by metabolic risks over the last three decades. The proportion of stroke deaths attributable to high SBP in those attributable to metabolic risk factors increased from 41.9 and 44.9% in 1990 to 48.9 and 49.8% in 2019 in men and women, respectively (Supplementary Figure A1). In contrast, the proportions of stroke deaths related to other metabolic risks changed modestly.

**Figure 1:**
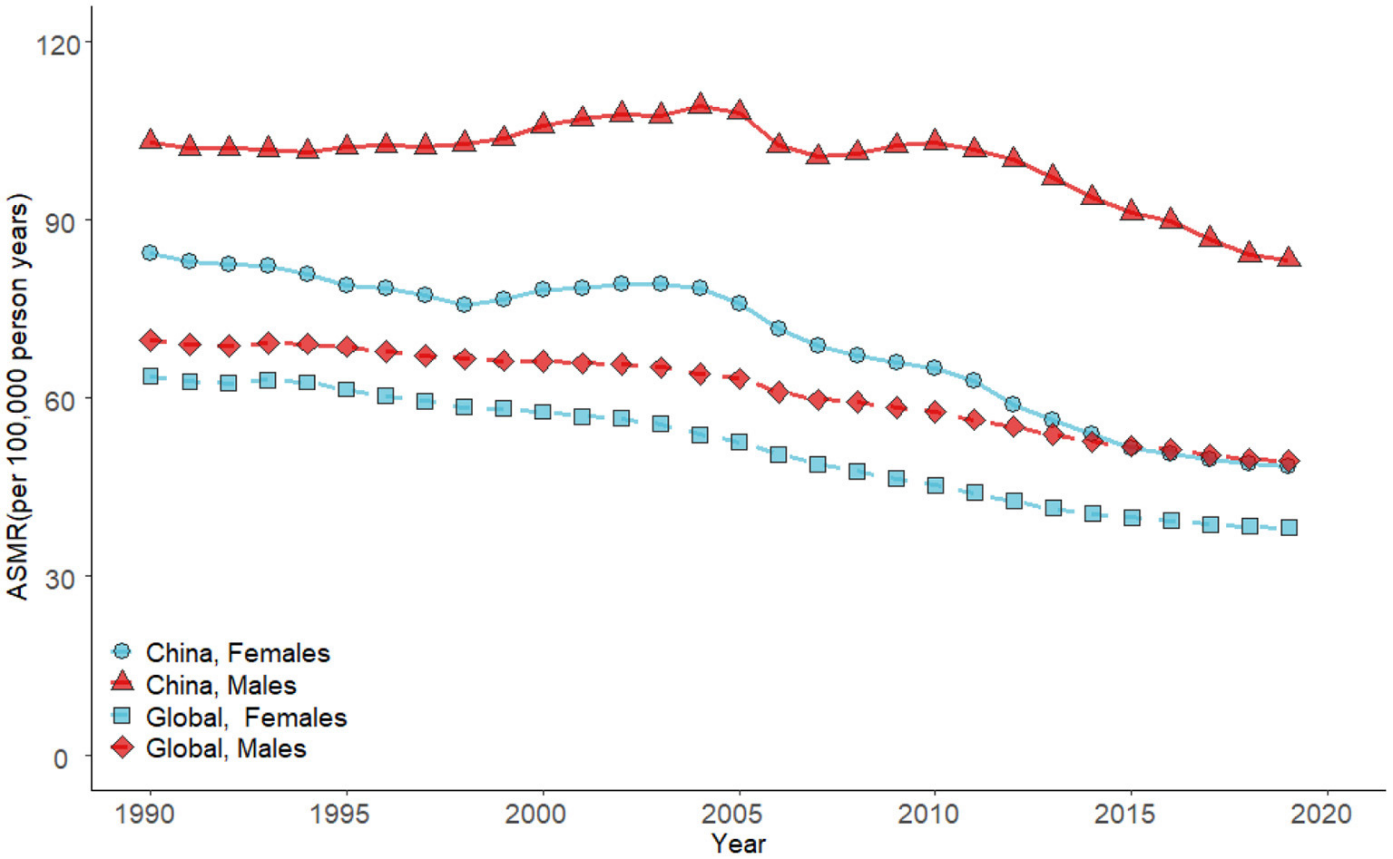
Trends in the ASMRs of stroke attributable to high SBP in China and global by gender from 1990 to 2019.

The age-standardized mortality rates (ASMRs) of stroke attributable to high SBP for both genders in China decreased significantly (Figure 1). The ASMRs per 100,000 decreased by 19.2% for men, from 103.2 in 1990 to 83.3 in 2019. In women, the ASMRs decreased by 42.6%, from 84.5 to 48.5 in China. However, the high SBP-attributable stroke mortality in China was higher than the global level. The ASMRs declined from 69.8 to 49.4 for men at the global level and also from 63.7 to 38.2 for women.

**Figure 2:**
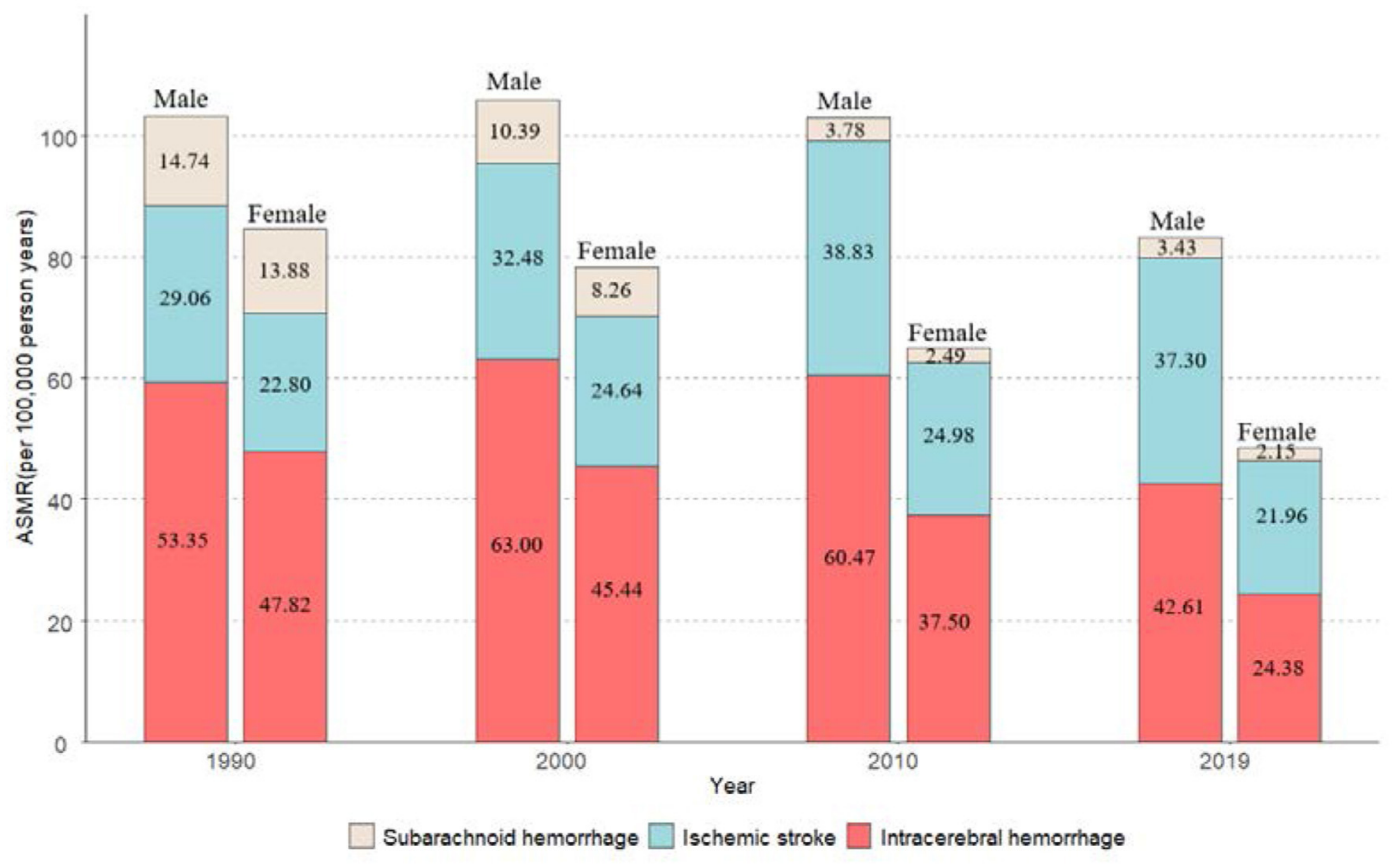
Trends in the ASMRs for subtypes of stroke attributable to high SBP by gender from 1990 to 2019 in China.

Figure 2 presents the ASMRs of stroke subtypes in China from 1990 to 2019. ICH mortality attributable to high SBP was the most prominent during the observation period (its proportion was over 50%). The second was IS; its mortality increased from 29.06 in 1990 to 37.30 in 2019 in men, while changing modestly in women, whereas SAH mortality attributable to high SBP was the least common among all stroke subtypes and declined successively. For gender difference, the mortalities of the above three subtypes were all slightly lower in women than those in men.

**Figure 3:**
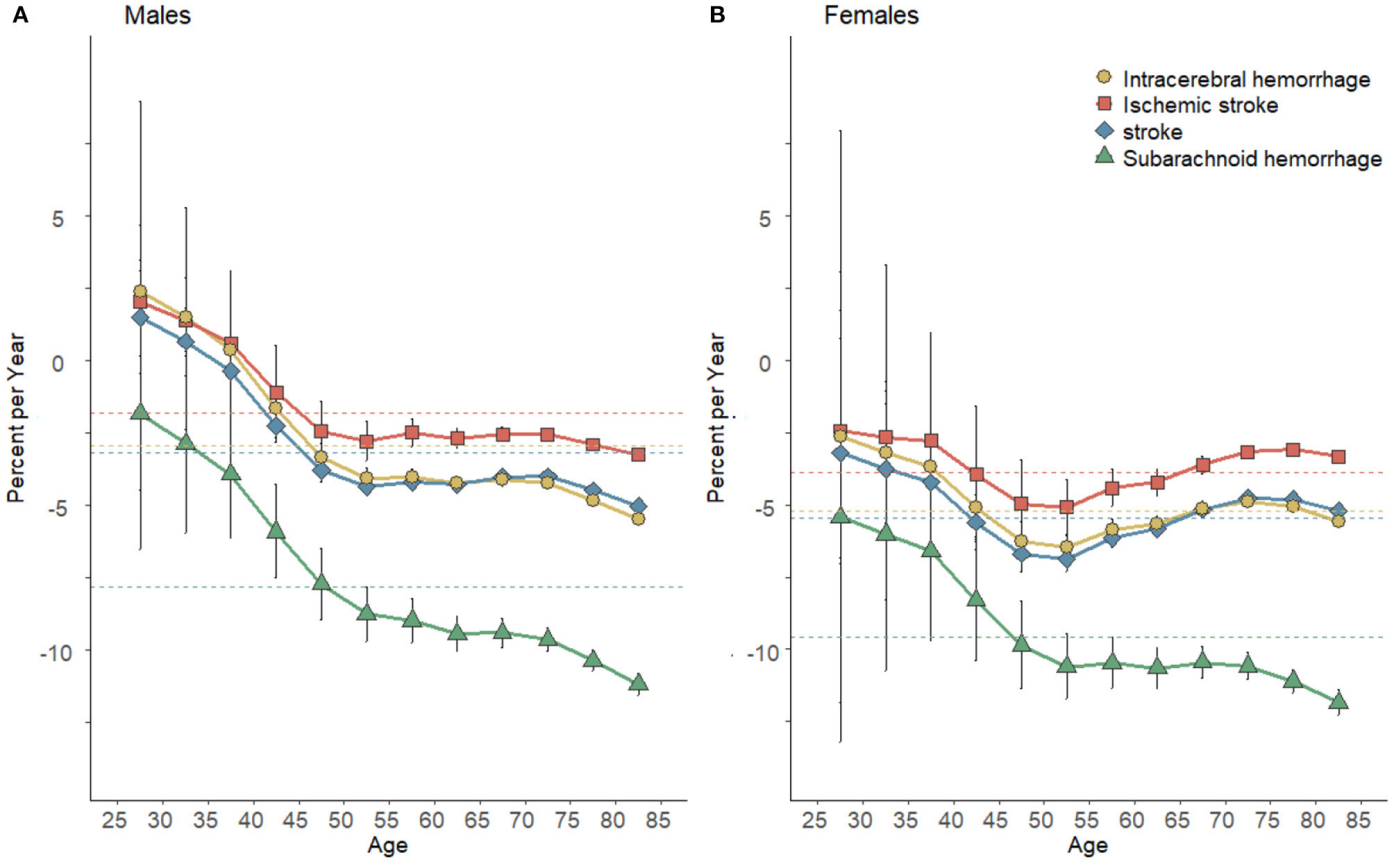
Local drift with net drift values for stroke mortality attributable to high SBP for men **(A)** and women **(B)** in China. Net drift expresses the overall annual percentage change, and the values <0, which indicated substantial reductions in high SBP-attributable stroke and subtype mortality across the study period. Net drifts express as colored dashed lines. Local drift values represent the annual percentage change in the respective age group. The local drift values <0, which revealed a decreasing trend of high SBP-attributable stroke and subtype mortality across the study period. Error bars represent the 95% CIs for the local drift values (some were too narrow to show).

Figure 3 illustrates the net drifts and local drifts for high SBP-attributable stroke and subtype mortality in C hina. Across the whole study period, the overall net drifts of stroke were higher in men (−3.19% [−4.42%, −2.97%]) than that of women (−5.45% [−5.85%, −5.04%]). For stroke subtypes, mortality reductions were the most striking in SAH (−7.81% [−8.43%, –7.19%] for men vs. −9.55% [−10.41%, −8.68%] for women) and the least striking in IS for both genders (−1.82% [−2.51%, −1.12%] for men vs. −3.86% [−4.87%, −2.85%] for women). The net drift of stroke and its subtypes in women were smaller than that in male counterparts, which indicated that the improvements in high SBP-attributable mortality are more prominent in women over the past three decades. All local drift values of stroke for women are lower than their counterparts of men. Although values were largely <0 for all age groups of women, the local drift values were fluctuated in stroke. Except for the continuous decline in high SBP-attributable SAH mortality, the decreasing trends of other subtypes slowed down among women aged over 55 years. The local drifts values were not constantly lower than 0 for men of stroke and its subtypes. Values across the age groups tended to decrease. SAH in both genders had the fastest decline in mortality among stroke subtypes, which revealed the improvements in controlling its mortality.

**Figure 4:**
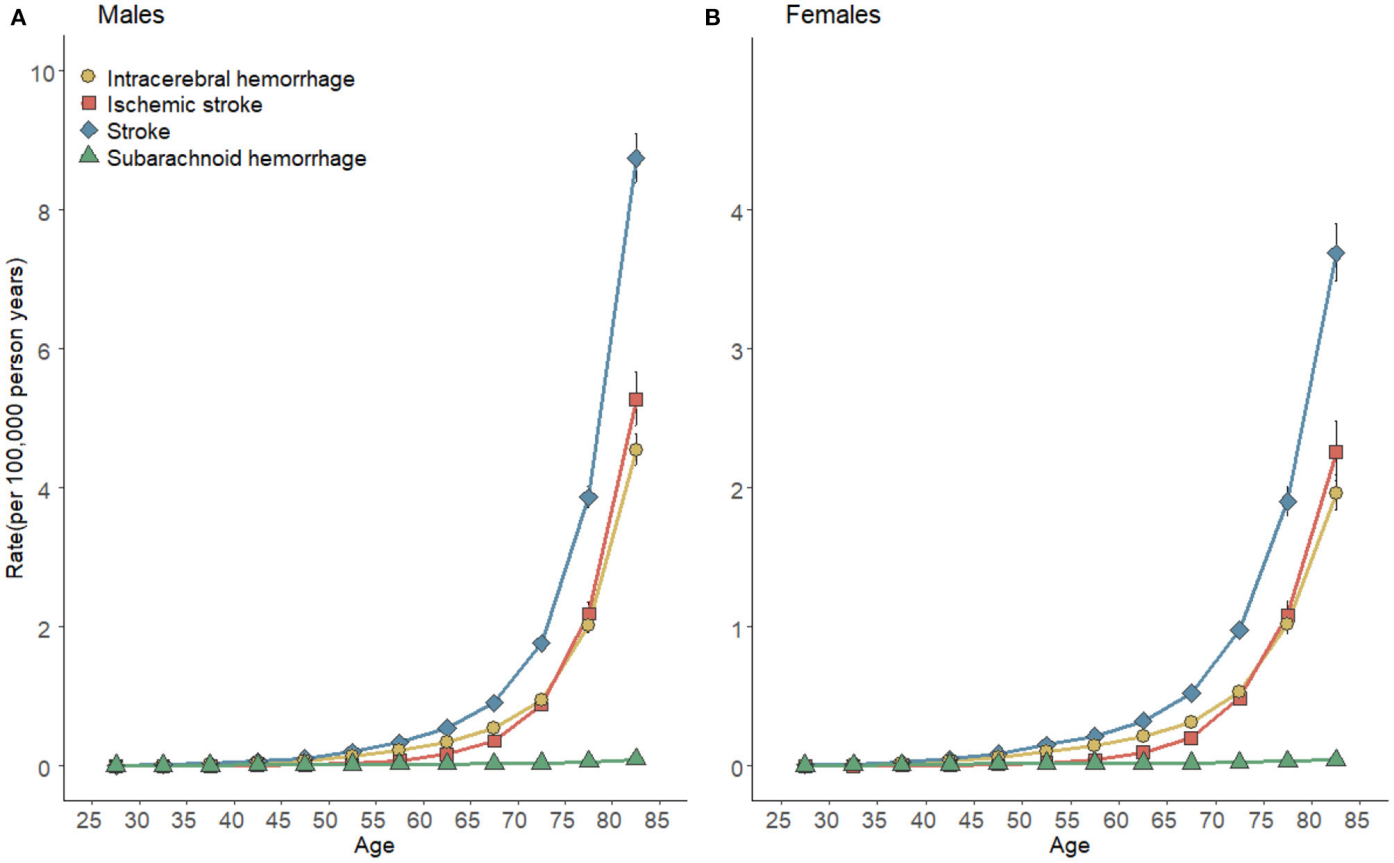
Longitudinal age curves of high SBP-attributable stroke mortality and subtypes for men **(A)** and women **(B)** in China. Fitted longitudinal age-specific rates of stroke mortality rates (per 100,000 person-years) and the corresponding 95% confidence intervals (some were too narrow to show).

Figure 4 plots the longitudinal age curves of high SBP-attributable stroke mortality rates by subtypes and gender. The above curves showed an exponential distribution (Table 1), thus indicating that the high SBP-attributable stroke and subtype mortality increased prominently with age and peak at the 80–84 age groups for both genders. The steepest increases with age were in high SBP-attributable ICH and IS, with almost the identical pace. As opposed to the above result, the rates of the high SBP-attributable SAH in both genders were kept at the bottom across all ages. The rates of stroke and all subtypes in men were slightly higher than women before 50 years old. However, after people were aged over 55 years, the increments of high SBP-attributable mortality in women would be lagged far behind men. After the adjustment for period deviations, curves of high SBP-attributable stroke can be expressed as *rate* = 0.004 × *e*^0.621 × *average age*^ (R-squared = 0.987) for high SBP-attributable stroke in men and *rate* = 0.005 × *e*^0.539 × *average age*^ (R-squared = 0.991) in women. The RR for increased high SBP-attributable stroke mortality at the respective life stage across each decade from age 24–29 to 80–84 were 2.08 for men and 1.09 for women.

**Table 1:** Estimated longitudinal age trend of high SBP-attributable stroke and subtypes mortality for men and women in China.

**Type of stroke**	**Curve equation**	**R-square**	**RR_**10**_**
**Men**			
Stroke	*y* = 0.004 × *e*^0.621 × *average age*^	0.987	2.080
ICH	*y* = 0.003 × *e*^0.596 × *average age*^	0.984	1.200
IS	*y* = 0.0002 × *e*^0.855 × *average age*^	0.999	1.030
SAH	*y* = 0.003 × *e*^0.277 × *average age*^	0.966	0.050
**Women**			
Stroke	*y* = 0.005 × *e*^0.539 × *average age*^	0.991	1.091
ICH	*y* = 0.003 × *e*^0.524 × *average age*^	0.990	0.638
IS	*y* = 0.0002 × *e*^0.755 × *average age*^	0.999	0.380
SAH	*y* = 0.004 × *e*^0.202 × *average age*^	0.930	0.030

*RR_10_ indicated the RRs for high SBP-attributable stroke and subtypes mortality every decade*.

*R-square indicated the goodness-of-fit of the curve equation; average age indicated the mean value of every age group*.

**Figure 5:**
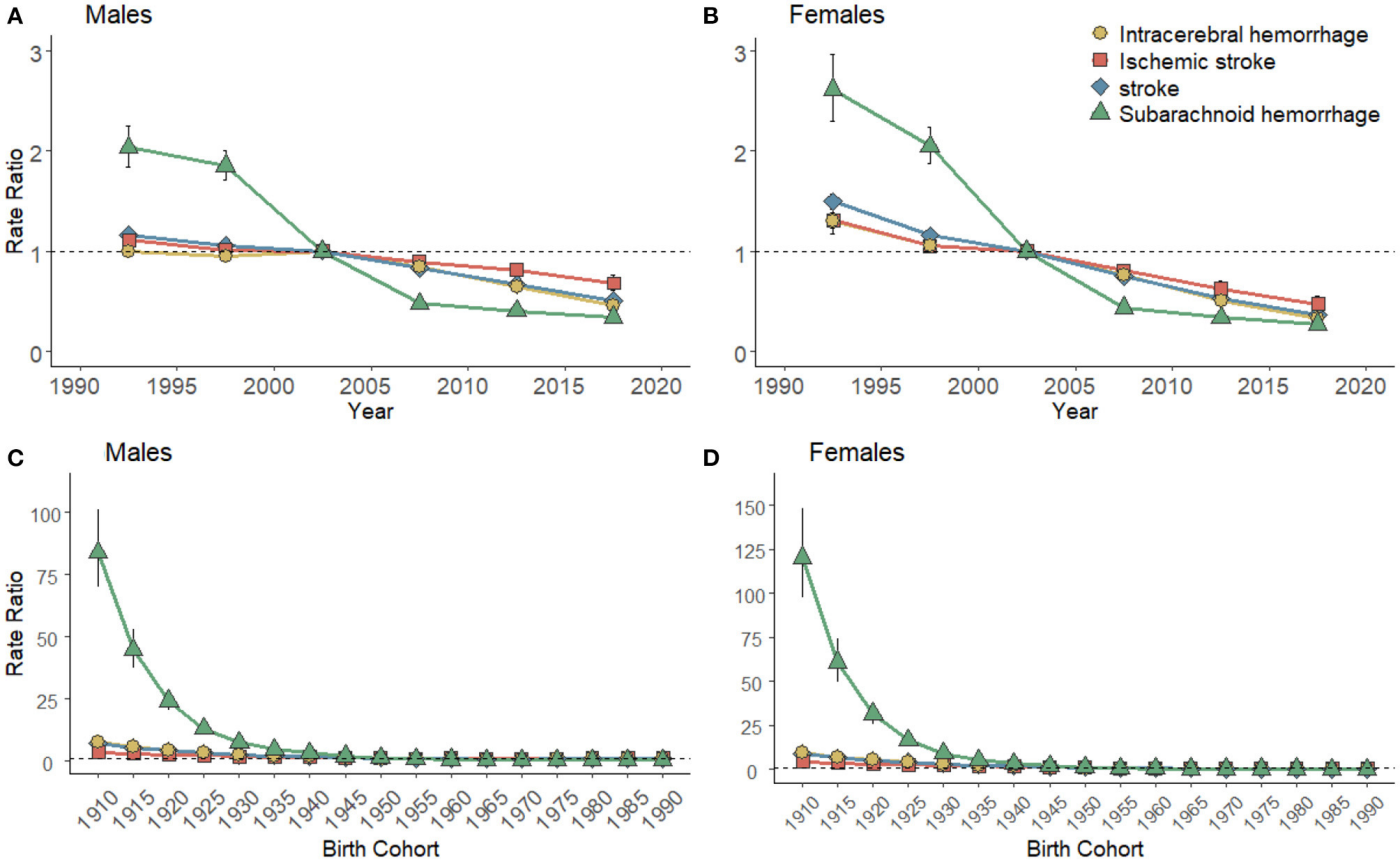
Period effects of high SBP-attributable stroke mortality for men **(A)** and women **(B)** in China. The RRs of the respective period compared with the reference period (2000–2005) adjusted for age and nonlinear cohort effects and the corresponding 95% CI. Cohort rate ratios of stroke mortality for men **(C)** and women **(D)** in China. The RRs of the respective cohort compared with the reference cohort (1945s) adjusted for age and nonlinear period effects and the corresponding 95% CI (some were too narrow to show).

Figure 5 illustrates the estimated period and cohort effects of the mortalities of high SBP-attributable stroke and subtypes by gender in China. The period effects on high SBP-attributable stroke and subtype mortality all showed a monotonic decreasing pattern for both genders (Figures 5A,B), with the most noticeable reductions for SAH (decreasing from 2.04 to 0.34 in men and 2.61 to 0.27 in women) over the past three decades. Likewise, the cohort effects of high SBP-attributable SAH decreased most significantly in successive birth cohorts (decreasing from 83.78 to 0.17 in men and from 120.11 to 0.05 in women). In contrast to the above result, the reductions in ICH (declining from 7.62 to 0.97 in men and from 9.76 to 0.18 women) and IS (declining from 3.37 to 1.00 in men and from 4.37 to 0.23 in women) were insignificant (Figures 5C,D). Though women had a greater risk of death in the earliest period or birth cohort, the same monotonic decline pattern was observed in men and women for period and cohort effects. Thus, it was revealed that women could show a more favorable trend than men.

Furthermore, as revealed by the results of the Wald test for the APC model (Table 2), the mortalities of stroke and subtypes attributable to high SBP showed a statistically significant difference in local drifts and net drifts, age, period, and cohort deviations represented the potential effects arising from age, cohort, and period on the observed temporal trends.

**Table 2:** Statistical parameters for overall and age-specific annual percent changes in age–period–cohort models.

**Diseases**	**Gender**	**All local drifts** **=** **net drift**		**Net drifts** **=** **0**		**All cohort deviations** **=** **1**		**All period deviations** **=** **1**	
		**Wald tests**	***p*-value**	**Wald tests**	***p*-value**	**Wald tests**	***p*-value**	**Wald tests**	***p*-value**
Stroke	Males	361.38	<0.01	736.89	<0.01	16,385.33	<0.01	1,248.30	<0.01
	Females	148.23	<0.01	671.64	<0.01	12,178.20	<0.01	940.37	<0.01
ICH	Males	369.3	<0.01	462.49	<0.01	9,962.67	<0.01	1,355.72	<0.01
	Females	94.58	<0.01	511.86	<0.01	8,564.73	<0.01	1,061.46	<0.01
IS	Males	53.92	<0.01	26.12	<0.01	2,483.30	<0.01	77.84	<0.01
	Females	38.22	<0.01	53.87	<0.01	2,252.19	<0.01	139.02	<0.01
SAH	Males	98.25	<0.01	562.44	<0.01	4993.08	<0.01	755.08	<0.01
	Females	35.63	<0.01	419.96	<0.01	4,330.20	<0.01	573.29	<0.01

## Discussions

As indicated by this article, stroke fatality attributed to high SBP has declined over the past three decades. In general, fewer fatality was reported in SAH arising from high SBP than IS and ICH in China in this period, which confirmed a decline of the burden of stroke deaths. There was a higher high SBP-related stroke death rate among the elders, according to the APC analysis. The period effects illustrated that the mortalities of all types of stroke have been decreasing between 1990 and 2019, with a leading role of decline in high SBP-attributable SAH. Furthermore, the reductions of SAH in the cohort effects proved more significant than other types. Thus, it was confirmed that the high SBP-related deaths for all types of stroke decreased in Chinese population, whereas the possible rebound should still be highlighted and worth attention.

Metabolic risk factors (high BMI, high FPG, high SBP, and high LDL cholesterol) were proved to be positively correlated with the occurrence and development of cardiovascular diseases (25, 26). Among all of them, high SBP always ranked 1st risk factor related to all types of stroke fatality from 1990 to 2019 (1). Suggested mechanisms mentioned by prior researches include the following: the elevation of SBP causes smooth muscle hypertrophy and remodeling that result in arterial wall thickness and lumen narrowing, enhancing endothelial dysfunction which increases vascular permeability that leads to local thrombi, and accelerating the atherosclerosis process in the intracerebral arteries by enhancing oxidative stress and inflammation that ends with vascular resistance (27, 28). The mentioned biological changes would be the dangerous inducements that could decrease the stroke outcome and cause of death. Thus, the effect arising from SBP change on the cerebral circulation is of high importance to the stroke burden.

Despite the period and cohort effects of high SBP-attributable stroke have been on a downward trajectory, it not easy to give a comprehensive interpretation separately. Since the mortalities of stroke and subtypes are highly dependent on the economic status and medical development, obviously, the period or group effect peaked among those born in 1990 and earlier. The abrupt declines in the risk of death for high SBP-attributable stroke indicated an enhanced individual health awareness, and behavior alternation was demonstrated as the primary preventive strategy to reduce high SBP (29). In recent years, it has become of high significance to eat less salt and more vegetables and fruits every day, plus enough physical activity. On the one hand, the Chinese government attempts to meet the goal that reduces the average salt per day from 12 g to <5 g (30, 31). Besides, China advances to meet the goals set by the Chinese Nutrition Society for alcohol consumption, smoking cession, daily fruit intake, regular physical activity, and maintaining healthy body weight, which could effectively help reduce high SBP-related stroke fatality or other severe outcomes (24, 32–35). On the other hand, the Chinese government strive to scale up urbanization and basic medical coverage to boost the availability, accessibility, and affordability of medical care, thus ensuring that more patients with potentially abnormal SBP are identified and given adequate interventions (36, 37). As indicated by the cohort effects, understanding biological significance of abnormal SBP under different historical backgrounds leads to the risk of deaths in difference (38). Younger generations have grown up in relatively beneficial circumstances, where good behavior and increased health awareness give them the opportunity to avoid the dangers of long-term exposure that can lead to high SBP (39). Suffering the lagged social economy and uncompleted healthcare system, older generations faced higher risk of deaths. Thus, the transformations of cohort effects are the bonus of the economic development and social progress.

Age has been found as the most influential demographic risk factor for stroke and its subtypes (40). Many previous studies confirmed that age was an independent and important risk factor for stroke, and the death caused by stroke varied across different age groups (24, 41, 42). Aging and population swell lead the proportion of Chinese stroke patients with high SBP as much as 84% (43). In this article, the risk of mortality for high SBP-attributable stroke keeps in exponential growth with age after adjusting for cohort or period deviations. There has been a consensus that the preventions or treatment strategies are less effective in older stroke patients than the young patients (44). As revealed by previous investigation, over 70% of deaths caused by stroke and its subtypes were reported in Chinese adults aged over 65 years, and over 80% of fatalities occurred in Chinese aged over 60 years (14). A prospective cohort study on a half million adults in China showed that SBP increased linearly with age in both sexes and was log-linearly related to stroke risk, and men more vulnerable than women at every age group (45). This article consolidates that older-aged population at higher mortality risks. Another topmost demographic risk factor is gender. Compared with women, men have higher ASMRs. Moreover, according to the APC model, the improvements in high SBP-attributable stroke mortality in women are more promising than those in men. The risk factors correlated with high SBP (e.g., alcohol use or tobacco) are more prevalent in men (12). The baseline mortality of stroke is higher in men since the above risk factors are positively correlated with high SBP and have interactive effects on high SBP (12, 46). Previous studies demonstrated a strong correlation between high SBP and stroke in women compared with men (47). It is therefore indicated that once women have their SBP under control, it can dramatically reduce the risk of high SBP-attributable stroke mortality. In fact, women are easy to acquire good behavior by interventions. Hence, the high SBP-attributable stroke mortality in women has better improvements than men.

In the analysis of this article, the temporal trends of various stroke subtype fatality are discrepant. Of note, the effect of high SBP on different classifications of stroke also varies. IS was suggested that mainly correlating with atherosclerosis, the formation of atherosclerosis has been significantly impacted by the increase of SBP and aging (48). High SBP also worsens SAH and SAH since the danger of brain tissue damage emerging from the rapture of cerebral arteries within or on the brain surface grows up with SBP (2). Lacey et al. (45) reported every 10 mmHg higher of usual SBP was correlated with a 30% higher risk of IS and 68% increased risk of ICH. ICH has traditionally lagged IS as far as life-threatening and disease burden; however, in contrast to IS, the incidence of ICH has been stable over the past few years (49, 50), probably because the high SBP-attributable ICH mortality was higher than that of the other stroke subtypes in China. Though the high SBP-attributable SAH mortality takes up the least share in all high SBP-related stroke deaths, the reductions of its period and cohort effects between 1990 and 2019 had been prominent. According to early research, the mortality of SAH in China was no longer lower than that in the west (51). Since SAH with a high fatality rate and younger mean age at onset can significantly impact public health, China has begun targeted preventions on a large scale over the last decades and finally reduced the burden of high SBP-attributable SAH greatly (51, 52). In brief, the declining trends of all stroke subtypes in China cannot be achieved without the adherence of their people to healthy behaviors besides the significant signs of progress in health indicators (e.g., healthcare coverage and advancement of the clinical diagnosis and treatment technology).

Nevertheless, this article still had some limitations. First, the data of this article originated from the latest GBD study, with certain deviations in the completeness and accuracy of stroke deaths. Although the GBD 2019 adopts numerous adjustments and corrections to the source, collation, and evaluation of the stroke mortality attribute to high SBP for improving data accuracy and comparability, it was indicated to be unlikely to completely reduce data inaccuracy. Second, mortality data of SAH and ICH attributable to high SBP aged younger than 25 and over 80 years were excluded from the analysis since the above data have been scarce in the GBD database. Third, consistent with other studies based on the population level, ecological fallacy might occur since the study has not focused on the individual level. Thus, subsequent studies should consider shortages in this article and avoid the above limitations.

## Conclusions

In summary, we estimated the long-term trends of stroke mortality attributable to high SBP by gender and stroke subtypes from 1990 to 2019. Also, this article assessed the contribution of age, period, and cohort effects to the trends. Although there were reductions in ASMRs for both genders over the past three decades in China, there has still been a gender gap in high SBP-attributable stroke. In general, men and elder groups were the high-risk populations for stroke mortality attributable to high SBP. For the independent period and cohort effects, a faster decreasing trend was observed in women than in men, whereas people born in the later birth cohorts or living in recent periods had lower risk of stroke death. Despite the high SBP-related stroke mortality in China keep declining, ICH and IS would continue to be the first and second lethal subtypes of stroke, respectively. As opposed to the above finding, SAH accounted for the lowest proportion of deaths and the greatest reduction in period and cohort effects. Thus, advanced technology on hypertension control approaches and knowledge regarding healthy lifestyles should be presented.

## Data Availability Statement

The datasets presented in this study can be found in online repositories. The names of the repository/repositories and accession number(s) can be found below: http://ghdx.healthdata.org/gbd-results-tool.

## Author Contributions

Conception and design of study, analysis and/or interpretation of data, and writing the original manuscript: CY and YM. Collating data and visualization: YM. Reviewing and editing the manuscript: YM, CY, JC, DY, JB, YZ, and QH. Funding acquisition: CY. All authors have read and agreed to the published version of the manuscript.

## Funding

This work was supported by the National Natural Science Foundation of China (Grant Numbers: 82173626 and 81773552).

## Conflict of Interest

The authors declare that the research was conducted in the absence of any commercial or financial relationships that could be construed as a potential conflict of interest.

## Publisher's Note

All claims expressed in this article are solely those of the authors and do not necessarily represent those of their affiliated organizations, or those of the publisher, the editors and the reviewers. Any product that may be evaluated in this article, or claim that may be made by its manufacturer, is not guaranteed or endorsed by the publisher.

## Abbreviations

SBP: Systolic blood pressure
IS: ischemic stroke
ICH: intracerebral hemorrhage
SAH: subarachnoid hemorrhage.

## References

[1] Roth GA Mensah GA Johnson CO Addolorato G Ammirati E Baddour LM. Global burden of cardiovascular diseases and risk factors, 1990-2019: update from the GBD 2019 study. J Am Coll Cardiol. (2020) 76:2982–3021. 10.1016/j.jacc.2020.11.010 33309175 PMC7755038

[2] Hisham NF Bayraktutan U. Epidemiology, pathophysiology, and treatment of hypertension in ischaemic stroke patients. J Stroke Cerebrovasc. (2013) 22:E4–E14. 10.1016/j.jstrokecerebrovasdis.2012.05.001 22682972

[3] Thorvaldsen P Asplund K Kuulasmaa K Rajakangas AM Schroll M. stroke incidence, case-fatality, and mortality in the who monica project. Stroke. (1995) 26:1503–4. 10.1161/01.STR.26.3.361 7886707

[4] Wu S Wu B Liu M Chen Z Wang W Anderson CS. Stroke in China: advances and challenges in epidemiology, prevention, and management. Lancet Neurology. (2019) 18:394–405. 10.1016/S1474-4422(18)30500-3 30878104

[5] G.B.D.R.F. Collaborators. Global burden of 87 risk factors in 204 countries and territories, 1990-2019: a systematic analysis for the Global Burden of Disease Study 2019. Lancet. (2020) 396:1223–49. 10.1016/S0140-6736(20)30752-2 33069327 PMC7566194

[6] A.J.H. Collaboration. Blood pressure indices and cardiovascular disease in the Asia Pacific Region - A pooled analysis. Hypertension. (2003) 42:69–75. 10.1161/01.HYP.0000075083.04415.4B 12756223

[7] Wright JT Jr. Bakris G Greene T Agodoa LY Appel LJ Charleston J. Hypertension study, effect of blood pressure lowering and antihypertensive drug class on progression of hypertensive kidney disease: results from the AASK trial. JAMA. (2002) 288:2421–31. 10.1001/jama.288.19.2421 12435255

[8] Van Gaal LF Mertens IL Block CEDe. Mechanisms linking obesity with cardiovascular disease. Nature. (2006) 444:875–80. 10.1038/nature05487 17167476

[9] Lawes CMM Vander Hoorn S Law MR Elliott P MacMahon S Rodgers A. Blood pressure and the global burden of disease 2000. Part II: Estimates of attributable burden. J Hypertens. (2006) 24:423–30. 10.1097/01.hjh.0000209973.67746.f0 16467640

[10] Yu X Tian X Wang S. J.J.o.t.L.S. Age-specific relevance of usual blood pressure to vascular mortality: a meta-analysis of individual data for one million adults in 61 propective studies. Lancet. (2002) 52:141–7. 10.1016/s0140-6736(02)11911-8 12493255

[11] Li Y Wang L Feng X Zhang M Huang Z Deng Q. Geographical variations in hypertension prevalence, awareness, treatment and control in China: findings from a nationwide and provincially representative survey. J Hypertens. (2018) 36:178–87. 10.1097/HJH.0000000000001531 29210864

[12] Lu JP Lu Y Wang XC Li XY Linderman GC Wu CQ. Prevalence, awareness, treatment, and control of hypertension in China: data from 1.7 million adults in a population-based screening study (China PEACE Million Persons Project). Lancet. (2017) 390:2549–58. 10.1016/S0140-6736(17)32478-9 29102084

[13] Cipolla MJ Liebeskind DS Chan SL. The importance of comorbidities in ischemic stroke: Impact of hypertension on the cerebral circulation. J Cereb Blood Flow Metab. (2018) 38:2129–49. 10.1177/0271678X18800589 30198826 PMC6282213

[14] Wang Z Hu S Sang S Luo L Yu C. Age-period-cohort analysis of stroke mortality in china: data from the global burden of disease study 2013. Stroke. (2017) 48:271–5. 10.1161/STROKEAHA.116.015031 27965429

[15] G.B.D. Mortality. Causes of death, global, regional, and national life expectancy, all-cause mortality, and cause-specific mortality for 249 causes of death, 1980-2015: a systematic analysis for the Global Burden of Disease Study 2015. Lancet. (2016) 388:1459–1544. 10.1016/S0140-6736(16)31012-1 27733281 PMC5388903

[16] Zhou M Wang H Zhu J Chen W Wang L Liu S. Cause-specific mortality for 240 causes in China during 1990-2013: a systematic subnational analysis for the Global Burden of Disease Study 2013. Lancet. (2016) 387:251–72. 10.1016/S0140-6736(15)00551-6 26510778

[17] Jha P Gajalakshmi V Gupta PC Kumar R Mony P Dhingra N. Study, Prospective study of one million deaths in India: Rationale, design, and validation results. PLoS Med. (2006) 3:191–200. 10.1371/journal.pmed.0030018 16354108 PMC1316066

[18] Burnett RT Pope CA 3rd Ezzati M Olives C Lim SS Mehta S . An integrated risk function for estimating the global burden of disease attributable to ambient fine particulate matter exposure. Environ Health Perspect. (2014) 122:397–403. 10.1289/ehp.1307049 24518036 PMC3984213

[19] Collaborators G. B.D.C.o.D. Global, regional, and national age-sex-specific mortality for 282 causes of death in 195 countries and territories, 1980-2017: a systematic analysis for the Global Burden of Disease Study 2017. Lancet. (2018) 392:1736–88. 30496103 10.1016/S0140-6736(18)32203-7 PMC6227606

[20] Rudd KE Johnson SC Agesa KM Shackelford KA Tsoi D Kievlan DR. Global, regional, and national sepsis incidence and mortality, 1990-2017: analysis for the Global Burden of Disease Study. Lancet. (2020) 395:200–11. 10.1016/S0140-6736(19)32989-7 31954465 PMC6970225

[21] Rosenberg PS Check DP Anderson WF. A web tool for age-period-cohort analysis of cancer incidence and mortality rates. Cancer Epidemiol Biomarkers Prev. (2014) 23:2296–302. 10.1158/1055-9965.EPI-14-0300 25146089 PMC4221491

[22] T.R. Holford. The estimation of age, period and cohort effects for vital rates. Biometrics. (1983) 39:311–24. 10.2307/2531004 6626659

[23] Zou Z Cini K Dong B Ma Y Ma J Burgner DP. Time trends in cardiovascular disease mortality across the BRICS: an age-period-cohort analysis of key nations with emerging economies using the global burden of disease study 2017. Circulation. (2020) 141:790–9. 10.1161/CIRCULATIONAHA.119.042864 31941371

[24] Cao J Eshak ES Liu K Gero K Liu Z Yu C. Age-period-cohort analysis of stroke mortality attributable to high sodium intake in china and japan. Stroke. (2019) 50:1648–54. 10.1161/STROKEAHA.118.024617 31195942 PMC6594775

[25] Hajjar I Kotchen TA. Trends in prevalence, awareness, treatment, and control of hypertension in the United States, 1988-2000. JAMA. (2003) 290:199–206. 10.1001/jama.290.2.199 12851274

[26] James PA Oparil S Carter BL Cushman WC Dennison-Himmelfarb C Handler J. 2014 evidence-based guideline for the management of high blood pressure in adults: report from the panel members appointed to the Eighth Joint National Committee (JNC 8). JAMA. (2014) 311:507–20. 10.1001/jama.2013.284427 24352797

[27] Shekhar S Liu R Travis OK Roman RJ Fan F. Cerebral autoregulation in hypertension and ischemic stroke: a mini review. J Pharm Sci Exp Pharmacol. (2017) 2017:21–7. 10.29199/JAPS.101013 29333537 PMC5765762

[28] Shekhar S Cunningham MW Pabbidi MR Wang S Booz GW Fan F. Targeting vascular inflammation in ischemic stroke: Recent developments on novel immunomodulatory approaches. Eur J Pharmacol. (2018) 833:531–44. 10.1016/j.ejphar.2018.06.028 29935175 PMC6090562

[29] Fan WG Xie F Wan YR Campbell NRC Su H. The impact of changes in population blood pressure on hypertension prevalence and control in China. J Clin Hypertens (Greenwich). (2020) 22:150–6. 10.1111/jch.13820 32003937 PMC8030006

[30] Du S Andrea N Carolina B Wang H Zhang B Zhang J. Understanding the patterns and trends of sodium intake, potassium intake, and sodium to potassium ratio and their effect on hypertension in China. Am J Clin Nutr. (2014) 99:334-43. 10.3945/ajcn.113.059121 PMC3893725 24257724

[31] Xi B Hao Y Liu F. Salt reduction strategies in China. Lancet. (2014) 383:1128. 10.1016/S0140-6736(14)60567-5 24679631

[32] Gu D He J Coxson PG Rasmussen PW Huang C Thanataveerat A. The cost-effectiveness of low-cost essential antihypertensive medicines for hypertension control in china: a modelling study. PLoS Med. (2015) 12:e1001860. 10.1371/journal.pmed.1001860 26241895 PMC4524696

[33] Luo L Jiang J Yu C Zhao M Wang Y Li Q. Stroke mortality attributable to low fruit intake in China: A joinpoint and age-period-cohort analysis. Front Neurosci. (2020) 14:552113. 10.3389/fnins.2020.552113 33335466 PMC7736244

[34] Roerecke M Kaczorowski J Tobe S Gmel G OJTLP Health. The effect of a reduction in alcohol consumption on blood pressure: a systematic review and meta-analysis. Lancet Public Health. (2017) 2:e108–20. 10.1016/S2468-2667(17)30003-8 29253389 PMC6118407

[35] Cao L Li X Yan P Wang X Yang K. J.J.o.C.H. The effectiveness of aerobic exercise for hypertensive population: A systematic review and meta-analysis. J Clin Hypertens. (2019) 21:868-76. 10.1111/jch.13583 PMC8030461 31169988

[36] Chaturvedi S. The seventh report of the joint national committee on prevention, detection, evaluation, and treatment of high blood pressure (JNC 7): is it really practical? Natl Med J India. (2004) 17:227. 15372777

[37] Lackland DT Carey RM Conforto AB Rosendorff C Whelton PK Gorelick PB. Implications of recent clinical trials and hypertension guidelines on stroke and future cerebrovascular research. Stroke. (2018) 49:772–9. 10.1161/STROKEAHA.117.019379 29467237 PMC5829017

[38] Yang Y. The intrinsic estimator for Age?eriod?ohort analysis: what it is and how to use it. Am J Soc. (2008) 113:6. 10.1086/587154

[39] Li J Li B Zhang F Sun Y. Urban and rural stroke mortality rates in China between 1988 and 2013: An age-period-cohort analysis. J Int Med Res. (2017) 45:680–90. 10.1177/0300060516664241 28415926 PMC5536664

[40] Griffiths D Sturm J. Epidemiology and etiology of young stroke. Stroke Res Treat. (2011) 2011:209370. 10.4061/2011/209370 21789269 PMC3140048

[41] Wang Y Peng Q Guo J Zhou LH Lu WL. Age-period-cohort analysis of type-specific stroke morbidity and mortality in China. Circ J. (2020) 84:662–9. 10.1253/circj.CJ-19-0803 32161200

[42] Sutton CJ Marsden J Watkins CL Leathley MJ Dey P. Changing stroke mortality trends in middle-aged people: an age-period-cohort analysis of routine mortality data in persons aged 40 to 69 in England. J Epidemiol Commun H. (2010) 64:523–9. 10.1136/jech.2008.086785 19822560

[43] Qureshi AI Ezzeddine MA Nasar A Suri MF Kirmani JF Hussein HM. Prevalence of elevated blood pressure in 563,704 adult patients with stroke presenting to the ED in the United States. Am J Emerg Med. (2007) 25:32–8. 10.1016/j.ajem.2006.07.008 17157679 PMC2443694

[44] Ekker MS Boot EM Singhal AB Tan KS Debette S Tuladhar AM. Epidemiology, aetiology, and management of ischaemic stroke in young adults. Lancet Neurol. (2018) 17:790–801. 10.1016/S1474-4422(18)30233-3 30129475

[45] Lacey B Lewington S Clarke R Kong XL Chen Y Guo Y. Age-specific association between blood pressure and vascular and non-vascular chronic diseases in 0·5 million adults in China: a prospective cohort study. Lancet Glob Health. (2018) 6:e641–e649. 10.1016/S2214-109X(18)30217-1 29773120 PMC5960069

[46] Gu D Reynolds K Wu X. J. Chen, He JJH. Prevalence, awareness, treatment, and control of hypertension in China. Hypertension. (2002) 40:920–7. 10.1161/01.HYP.0000040263.94619.D5 12468580

[47] Madsen TE Howard G Kleindorfer DO Furie KL Oparil S Manson JE. Sex differences in hypertension and stroke risk in the regards study: a longitudinal cohort study. Hypertension. (2019) 74:749–55. 10.1161/HYPERTENSIONAHA.119.12729 31405299 PMC6741430

[48] Bamford J Sandercock P Dennis M Burn J Warlow C. Classification and natural history of clinically identifiable subtypes of cerebral infarction. Lancet. (1991) 337:1521–6. 10.1016/0140-6736(91)93206-O 1675378

[49] Hemphill JC 3rd Greenberg SM Anderson CS Becker K Bendok BR Cushman M. Guidelines for the management of spontaneous intracerebral hemorrhage: a guideline for healthcare professionals from the american heart association/american stroke association. Stroke. (2015) 46:2032–60. 10.1161/STR.0000000000000069 26022637

[50] Flaherty ML Woo D Haverbusch M Sekar P Khoury J Sauerbeck L. Racial variations in location and risk of intracerebral hemorrhage. Stroke. (2005) 36:934–7. 10.1161/01.STR.0000160756.72109.95 15790947

[51] McGurgan IJ Clarke R Lacey B Kong XL Chen Z Chen Y. Blood pressure and risk of subarachnoid hemorrhage in China. Stroke. (2018) STROKEAHA118022239. 10.1161/STROKEAHA.118.022239 PMC6314500 30580702

[52] Ingall T Asplund K Mahonen M Bonita R. A multinational comparison of subarachnoid hemorrhage epidemiology in the WHO MONICA stroke study. Stroke. (2000) 31:1054–61. 10.1161/01.STR.31.5.1054 10797165

