# Supplementary material for: Age–Period–Cohort Analysis of Long Trend of Mortality for Stroke and Subtypes Attributed to High SBP in Chinese Adults
Source: Front Neurol. 2022 Mar 9;13:710744. doi: 10.3389/fneur.2022.710744 (PMC8959307; doi:10.3389/fneur.2022.710744)
Supplement: Supplementary file 1 [file Image_1.pdf]

Supplementary Material

1 Supplementary Figures and Tables

1.1 Supplementary Figures

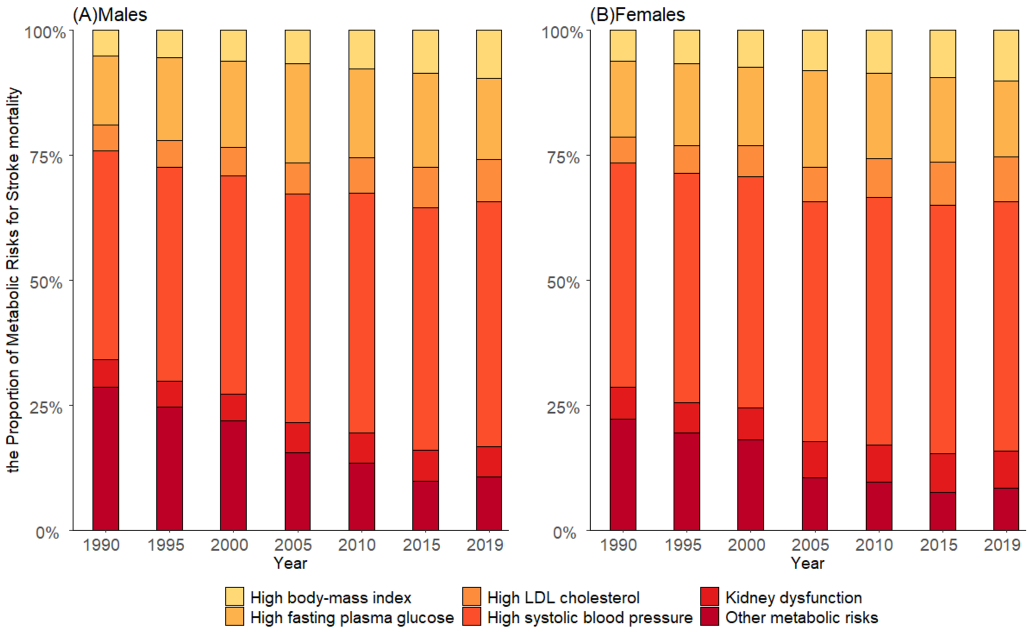

Supplementary Figure 1. The proportions of stroke mortality attributed to six metabolic risks for males(A) and females in China from 1990 to2019 (B).
